# Glycogen synthase kinase-3 controls IL-10 expression in CD4^+^ effector T-cell subsets through epigenetic modification of the IL-10 promoter

**DOI:** 10.1002/eji.201444661

**Published:** 2015-02-17

**Authors:** Elaine V Hill, T H Sky Ng, Bronwen R Burton, Charly M Oakley, Karim Malik, David C Wraith

**Affiliations:** 1 School of Cellular and Molecular Medicine, University of BristolBristol, UK; 2 Cancer Epigenetics Lab, School of Cellular and Molecular Medicine, University of BristolBristol, UK

**Keywords:** CD4^+^ T cells, Epigenetic, Glycogen synthase kinase-3, IL-10

## Abstract

The serine/threonine kinase glycogen synthase kinase-3 (GSK3) plays an important role in balancing pro- and anti-inflammatory cytokines. We have examined the role of GSK3 in production of IL-10 by subsets of CD4^+^ T helper cells. Treatment of naive murine CD4^+^ T cells with GSK3 inhibitors did not affect their production of IL-10. However, treatment of Th1 and Th2 cells with GSK3 inhibitors dramatically increased production of IL-10. GSK3 inhibition also led to upregulation of IL-10 among Th1, Th2, and Th17 subsets isolated from human blood. The encephalitogenic potential of GSK3 inhibitor treated murine Th1 cells was significantly reduced in adoptive transfer experiments by an IL-10-dependent mechanism. Analysis of the murine IL-10 promoter in response to inhibition of GSK3 in Th1 cells showed modification to a transcriptionally active state indicated by changes in histone H3 acetylation and methylation. Additionally, GSK3 inhibition increased expression of the transcription factors c-Maf, Nfil3, and GATA3, correlating with the increase in IL-10. These findings are important in the context of autoimmune disease since they show that it is possible to reprogram disease-causing cells through GSK3 inhibition.

## Introduction

IL-10 is essential for protection from immunopathology, allergy, and autoimmunity and is expressed by a wide variety of innate and adaptive immune cells 1,2. IL-10 production by Th1 cells is important for their self-regulation, to limit the immune response and prevent tissue damage in both infection and autoimmune disease 3–5. In the Tg4 TCR-transgenic mouse model, repeated administration of the Ac1-9 peptide of myelin basic protein (MBP) leads to induction of Th1 cells secreting IL-10 that protect mice from experimental autoimmune encephalomyelitis (EAE) 6. IL-10 secreted by these cells acts on dendritic cells (DCs) and renders them less effective at priming CD4^+^ T cells and suppresses their differentiation into Th1 cells, thus creating a negative feedback loop to prevent excessive Th1 inflammation 6. Th17 cells can also express IL-10, which is enhanced in the absence of IL-23 7. Th2 cells provide a protective response during parasite infection but are also involved in allergic responses through the enhancement of IgE induction. IL-10 secretion by Th2 cells is important in restraining Th2 responses in murine allergy 8 and Th2-derived IL-10 can act on DCs to prevent further differentiation of Th2 cells 9.

The serine/threonine kinase glycogen synthase kinase-3 (GSK3) has been shown to have an important role in regulating IL-10 expression 10,11. Inhibitors of GSK3 have been shown to reduce inflammation in experimental colitis, arthritis, and peritonitis 12,13; they also led to downregulation of pro-inflammatory cytokines and upregulation of IL-10 in a model of endotoxin shock 14. GSK3 inhibition in human memory CD4^+^ T cells, but not naive cells, was found to increase IL-10 production and IL-10-dependent suppressive activity 15.

Lithium is an inhibitor of GSK3 that has been used to treat bipolar disorder in humans for over 50 years 16. A study treating C57BL/6 mice with dietary lithium suppressed EAE both prior to and after disease induction 17. Furthermore, the generation of Th1 cells was reduced by GSK3 inhibition, due to impaired STAT1 activation 18, while inhibition of GSK3 in CD4^+^ T cells led to a block in IL-6 production and STAT3 activation, thereby preventing Th17 polarization 19.

In this study, we investigated whether GSK3 inhibition affects IL-10 production in different subsets of mouse and human CD4^+^ T cells. While inhibition of GSK3 did not affect IL-10 production in naive cells, treatment of Th1, Th2, or Th17 cells led to an increase in IL-10. Epigenetic changes at the IL-10 locus and IL-10-promoting transcription factors were induced by GSK3 inhibition of Th1 and Th2 cells leading to the generation of a nonpathogenic T-cell phenotype. We conclude that GSK3 controls the balance of pro- and anti-inflammatory cytokines in activated CD4^+^ T cells and that inhibition of GSK3 may have therapeutic utility in conversion of pathogenic CD4^+^ effector T cells into IL-10-secreting CD4^+^ T cells.

## Results

### GSK3 inhibition leads to increased IL-10 production by Th1, Th2, and Th17 cells

**Figure 1:**
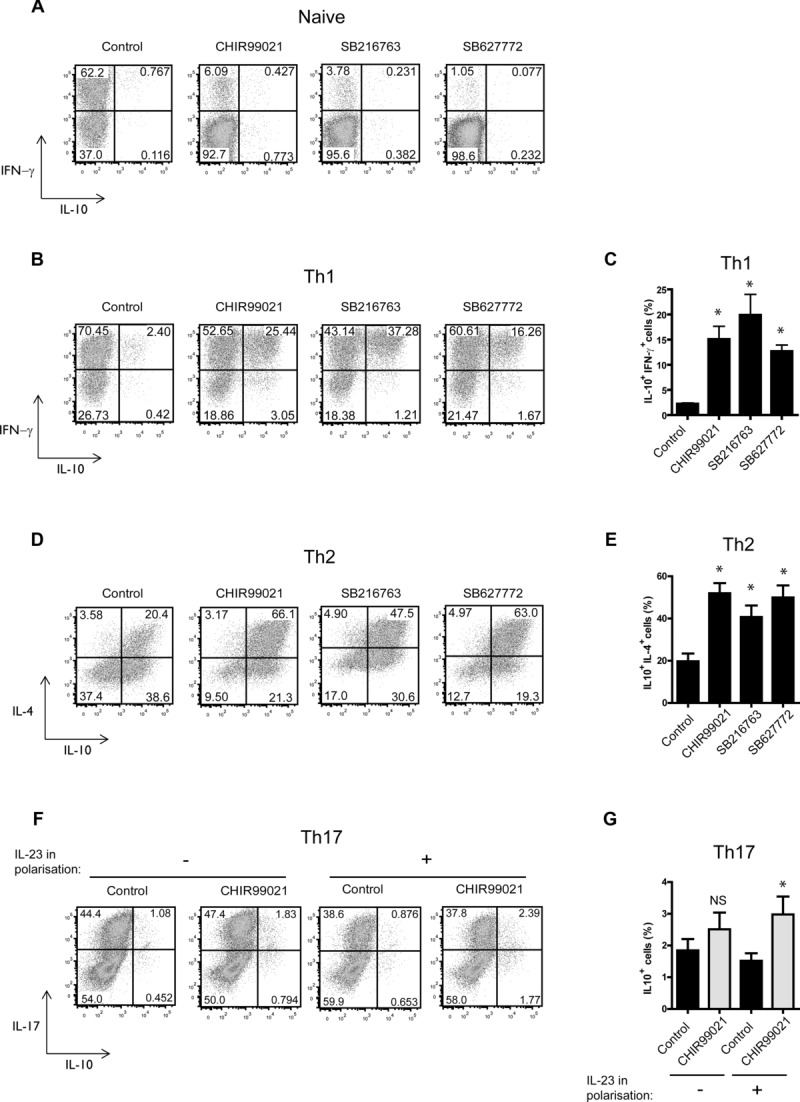
GSK3 inhibitors induce IL-10 production in Th1, Th2, and Th17 cells. (A) Naive CD4^+^ T cells were isolated from Tg4 splenocytes and cultured with APCs in the presence of MBP Ac1-9 and GSK3 inhibitors or vehicle control. Intracellular cytokine staining for IFN-γ and IL-10 was carried out on day 7 of stimulation. Data are plots gated on live CD4^+^ cells and are representative of three independent experiments. (B) Th1 polarized Tg4 cells were restimulated with APCs in the presence of GSK3 inhibitors or vehicle control and MBP Ac1-9. IL-2 was added on day 3 of culture and intracellular cytokine staining for IFN-γ and IL-10 was carried out on day 7. Data are plots gated on live CD4^+^ cells and are representative of five independent experiments. (C) Pooled quantitation of IL-10^+^/ IFN-γ^+^ cells from five independent experiments as shown in (B). Data are mean + SEM. *p < 0.05 (ANOVA, Dunnett's multiple-comparison posttest). (D) Th2 polarized Tg4 cells were restimulated with APCs in the presence of GSK3 inhibitors or vehicle control and MBP Ac1-9. IL-2 was added on day 3 of culture and intracellular cytokine staining for IL-4 and IL-10 carried out on day 7. Data are plots gated on live CD4^+^ cells and are representative of four independent experiments. (E) Pooled quantitation of IL-10^+^/ IL-4^+^ cells from four independent experiments as shown in (D). Data are mean + SEM. *p < 0.05 (ANOVA; Dunnett's multiple-comparison posttest). (F) Tg4 splenocytes were cultured under Th17 polarizing conditions with/without IL-23 added on day 3 of culture. On day 7 of culture, Th17 cells were restimulated with APCs in the presence of CHIR99021 or vehicle control and MBP Ac1-9. Intracellular cytokine staining for IL-17 and IL-10 was carried out on day 7 of stimulation. Data are plots gated on live CD4^+^ cells and are representative of six independent experiments. (G) Pooled quantitation of IL-10^+^ cells from six independent experiments as shown in (F). Data are mean + SEM. NS, not significant; *p < 0.05 (Student's *t*-test).

Naive CD4^+^ T cells were purified from spleens of Tg4 mice that express TCR specific for the peptide Ac1-9 of MBP and cultured with Ag-presenting cells (APCs) and peptide. These cells did not show any change in IL-10 production when cultured in the presence of GSK3 inhibitors although there was a decrease in the percentage of IFN-γ^+^ cells (Fig. 1A). We used three ATP-competitive inhibitors, CHIR99021, SB216763, and SB627772, with differing chemical structures and specificity profiles 20,21 in order to minimize off-target effects. To assess the effect of GSK3 inhibitors on effector T-cell subsets, Tg4 CD4^+^ T cells were polarized to a Th1 phenotype and then stimulated with APCs in the presence of GSK3 inhibitors. There was an increase in the percentage of cells producing IL-10 in cultures treated with GSK3 inhibitors (Fig. 1B). There was a significant increase in the population of IL-10^+^/IFN-γ^+^ cells (Fig. 1C). Similar results were observed with the peptide substrate competitive GSK3 inhibitor L803mts that has previously been shown not to inhibit other protein kinases 22, 23] (Supporting Information Fig. 1). Unlike the naive cells, there was no overall decrease in IFN-γ^+^ cells observed by intracellular cytokine staining of Th1 cells, however, there was a decrease in the amount of IFN-γ secreted by Th1 cells in an APC-independent system (Supporting Information Fig. 2); therefore, GSK3 inhibition is able to reduce IFN-γ expression in both naive and Th1 cells. There was an increase in polarized Th2 cells producing IL-10 in response to GSK3 inhibition (Fig. 1D) and there was a particularly large increase in cells expressing both IL-10 and IL-4 (Fig. 1E). Culture of polarized Th17 cells with CHIR99021 also led to a small but significant increase in the percentage of IL-10-expressing cells in cultures where IL-23 was included during polarization (Fig. 1F and G).

GSK3 activity in Th1 and Th2 cells cultured with GSK3 inhibitors was assessed by Western blotting of P-CRMP2 (T514), a GSK3 substrate 24, and this confirmed that GSK3 is inhibited throughout the culture (7 days) with the GSK3 inhibitors (Supporting Information Fig. 3). The effect of GSK3 inhibitors on IL-10 production during polarization of Th1, Th2, and Th17 cells was also studied (Supporting Information Fig. 4). There was a small increase in IL-10 production in Th1 and Th17 polarizing cells and a larger increase in IL-10 in Th2 polarizing cells; interestingly this was mainly from non-IL-4-expressing cells, unlike established polarized Th2 cells where the increase was predominantly in IL-4^+^IL-10^+^ cells (Fig. 1D). We also investigated the effect of GSK3 inhibition on the restimulation of cells cultured with peptide but no polarizing cytokines. These cells are mainly of a Th1 nature at day 7 and upon restimulation with GSK3 inhibitors increase production of IL-10 (Supporting Information Fig. 5).

IL-10 has previously been shown to promote anergy and the differentiation of IL-10-secreting Th1 cells 25,26. However, during the induction of IL-10 Tregs in vivo by repeated doses of peptide in the Tg4 mouse, exogenous IL-10 from sources such as APCs is not required 6. To ascertain whether the same is true in the conversion of Th1 cells to IL-10-producing cells by GSK3 inhibitors, Th1 cells were cultured with APCs from *IL-10*^−/−^ mice in the presence of GSK3 inhibitors. APC-derived IL-10 was not necessary for the increased production of IL-10 from CD4^+^ T cells following GSK3 inhibition; in fact, exogenous IL-10 partially suppressed IL-10 secretion (Supporting Information Fig. 6A and B). In similar experiments carried out in Th2 cells, exogenous IL-10 from APCs was also redundant for GSK3 inhibition mediated IL-10 induction (Supporting Information Fig. 6C and D).

In order to assess the requirement for TCR signaling in the upregulation of IL-10, Th1 and Th2 cells were restimulated with irradiated APCs in the absence of peptide (Supporting Information Fig. 7A and B). These cells did not increase their production of IL-10, showing the dependence on TCR signaling for this upregulation. We also assessed the requirement for the major Th1 and Th2 cytokines, IFN-γ, and IL-4 and IL-10 signaling by the addition of blocking antibodies to cultures of Th1 and Th2 cells with GSK3 inhibitor (Supporting Information Fig. 7C and D). Blocking of these cytokines did not affect the upregulation of IL-10.

### GSK3 inhibitors act directly on CD4^+^ T cells to induce IL-10 production in Th1, Th2, and Th17 cells

**Figure 2:**
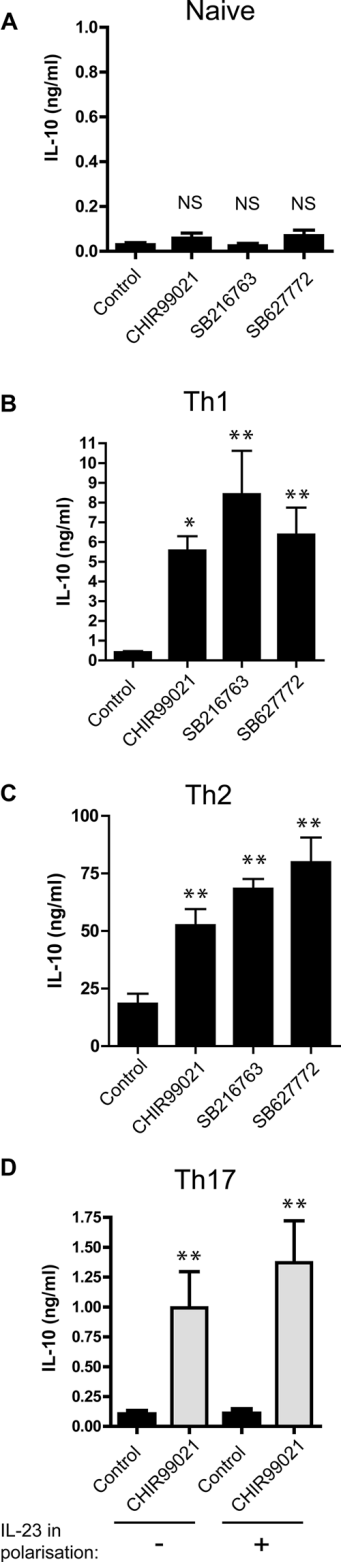
GSK3 inhibitors act directly on CD4^+^ T cells to induce IL-10 production in Th1, Th2, and Th17 cells. (A) Naive CD4^+^ T cells were isolated from Tg4 splenocytes and cultured with anti-CD3 and anti-CD28 in the presence of IL-2 and GSK3 inhibitors or vehicle control. Tissue culture supernatants were taken on day 7 of stimulation and analyzed for IL-10 by fluorescent bead immunoassay. Data are mean + SEM pooled from three independent experiments with triplicate wells. NS, not significant (ANOVA). (B) Th1 polarized Tg4 cells were restimulated with anti-CD3 and anti-CD28 in the presence of IL-2 and GSK3 inhibitors or vehicle control. Tissue culture supernatants were taken on day 7 of stimulation and analyzed for IL-10 by ELISA. Data are mean + SEM pooled from five independent experiments with triplicate wells. *p < 0.05, **p < 0.01 (ANOVA, Dunnett's multiple-comparison posttest). (C) Th2 polarized Tg4 cells were restimulated with anti-CD3 and anti-CD28 in the presence of IL-2 and GSK3 inhibitors or vehicle control. Tissue culture supernatants were taken on day 7 of stimulation and analyzed for IL-10 by ELISA. Data are mean + SEM pooled from five independent experiments with triplicate wells. **p < 0.01 (ANOVA, Dunnett's multiple-comparison posttest). (D) Tg4 splenocytes were cultured under Th17 polarizing conditions with/without addition of IL-23 on day 3. On day 7 of culture, Th17 CD4^+^ cells were isolated and restimulated with anti-CD3 and anti-CD28 in the presence of CHIR99021 or vehicle control. Tissue culture supernatants were taken on day 7 of stimulation and IL-10 was quantified by ELISA. Data are mean + SEM pooled from five independent experiments with triplicate wells. **p < 0.01 (ANOVA, Dunnett's multiple-comparison posttest).

To investigate the requirement for APCs during GSK3 inhibition mediated induction of IL-10 in CD4^+^ T cells, naive or polarized Tg4 CD4^+^ T cells were cultured with anti-CD3/ anti-CD28 stimulation in the presence of GSK3 inhibitors and IL-10 secretion was assessed. Naive CD4^+^ T cells did not show any increase in IL-10 secretion (Fig. 2A); however, both Th1 and Th2 cultures showed a substantial increase in IL-10 production (Fig. 2B and C), proving that GSK3 inhibitors act directly on the polarized CD4^+^ Th1 and Th2 cells. Polarized Th17 cells, cultured with GSK3 inhibitors, also upregulated IL-10 secretion (Fig. 2D) both with and without IL-23 during Th17 differentiation although the total IL-10 secreted was much lower than in murine Th1 or Th2 cells.

### GSK3 inhibition causes IL-10 upregulation in human Th1, Th2, and Th17 cells

**Figure 3:**
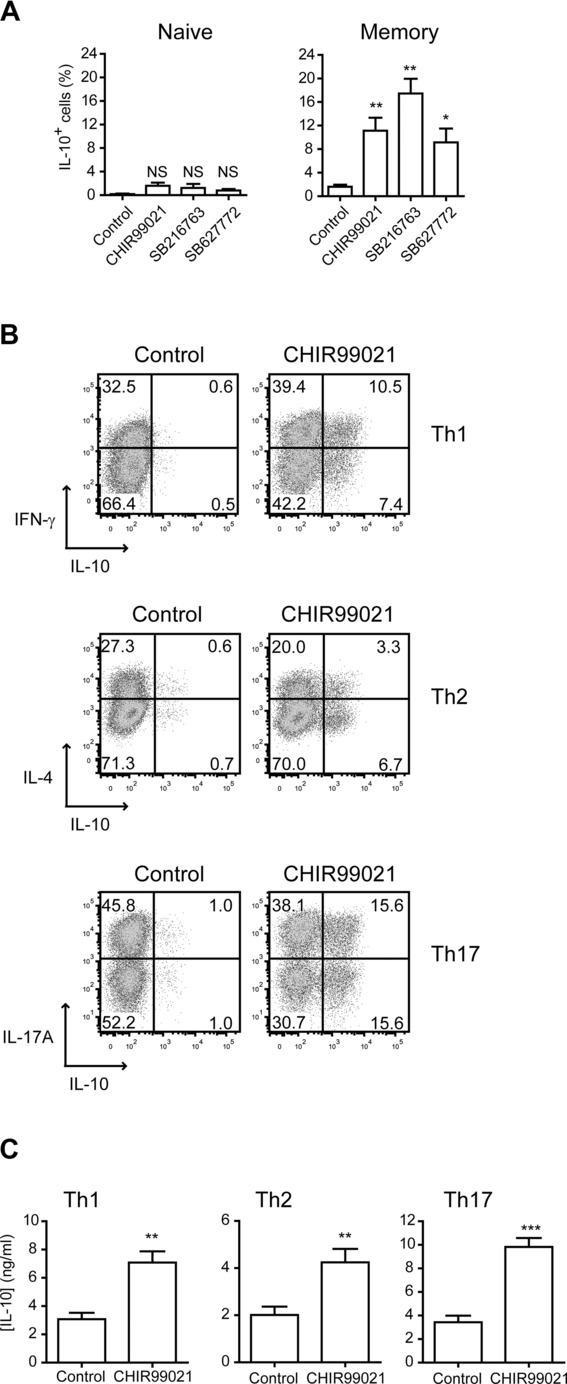
GSK3 inhibitors induce IL-10 production in human Th1, Th2, and Th17 cells. (A) Naive or memory CD4^+^ T cells were isolated from PBMCs by magnetic selection and stimulated with anti-CD3 and anti-CD28 in the presence of IL-2 and CHIR99021, SB216763, SB627772, or vehicle control. Intracellular cytokine staining was carried out on day 10. Graphs show the percentage (mean + SEM) of viable CD4^+^ T cells that are IL-10^+^ in each condition (naive *n* = 4, memory *n* = 10 donors). NS, not significant; *p < 0.05, **p < 0.01 (ANOVA, Dunnett's multiple-comparison posttest). (B) Human effector CD4^+^ T-cell subsets were isolated from PBMCs by flow cytometric sorting. Th1 cells (CD4^+^CXCR3^+^), Th2 cells (CD4^+^CXCR3^−^CCR4^+^CCR6^−^), or Th17 cells (CD4^+^CXCR3^−^CCR4^+^CCR6^+^) were cultured with autologous APCs, anti-CD3, IL-2, and CHIR99021 or vehicle control. Intracellular cytokine staining was carried out on day 7. Dot plots show cytokine production by live CD4^+^ cells. Data are representative of three independent experiments. (C) Tissue culture supernatants were taken on day 2 of culture and analyzed for IL-10 by fluorescent bead immunoassay. Data show mean + SEM of three pooled independent experiments. **p < 0.01, ***p < 0.001 (*t*-test).

We extended our studies to human CD4^+^ T cells and found that although naive cells showed a small, nonsignificant trend toward increased IL-10, memory cells responded with a significant increase in the percentage of cells expressing IL-10 (Fig. 3A). Cell sorting based on chemokine receptor expression, as described previously 22,23, enriched populations of IFN-γ-producing Th1 cells, IL-4-secreting Th2 cells, and IL-17A-expressing Th17 cells from human blood (Supporting Information Fig. 8). These purified subsets were cultured with polyclonal stimulation in the presence of CHIR99021, the most specific commercially available GSK3 inhibitor 20. Similar to our findings in the mouse, IL-10 was upregulated in each of the human subsets examined (Fig. 3B and C).

### GSK3 inhibition reduces the pathogenicity of Th1 cells

**Figure 4:**
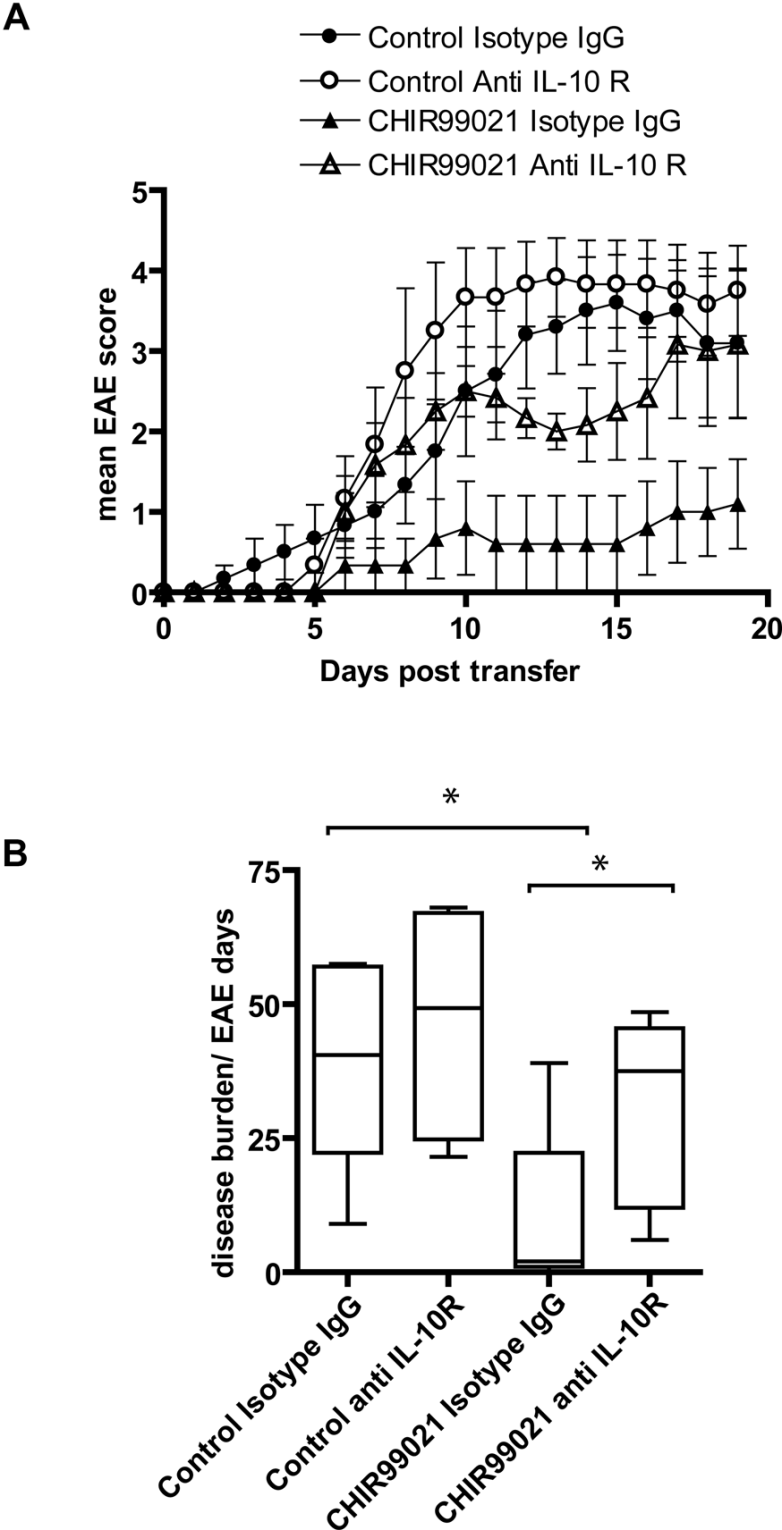
GSK3 inhibition abrogates the ability of Th1 cells to induce EAE in an IL-10-dependent manner. Th1 polarized Tg4 cells were restimulated with APCs and MBP Ac1-9 in the presence of CHIR99021 or vehicle control. IL-2 was added on day 3 of restimulation and on day 4, 10^7^ Th1 cells were adoptively transferred to Tg4 mice by i.p. injection. Recipient mice were also injected with anti-IL-10R Ab or isotype control (500 μg) on the day of transfer (day 0) and on day 7. EAE was scored daily. Three similar experiments were performed. Each group contained five to six mice. (A) Time course of disease progression. Data show the mean EAE score and SEM from each day. (B) Disease burden in EAE days was calculated for each mouse (median; error bars show minimum and maximum disease burden in each group; box extends from the 25th to 75th percentiles). The median score for the control isotype IgG group was significantly different from that of the CHIR99021 isotype IgG group and the CHIR99021 isotype IgG group was significantly different from that of the CHIR99021 anti-IL-10R group. *p < 0.05 (Mann–Whitney *U* test).

IL-10 has previously been shown to mediate protection from EAE when tolerance was induced in mice by repeated intranasal doses of peptide 27. We, therefore, tested whether the increased secretion of IL-10 from GSK3 inhibitor treated Th1 cells would have a protective role in EAE. Th1 cells were cultured +/- GSK3 inhibitor and adoptively transferred to Tg4 mice to induce EAE. Culture of Th1 cells with GSK3 inhibitor resulted in a decrease in EAE severity, which was sustained for the entire disease course (Fig. 4A). Mice receiving a control isotype Ab showed a significant decrease in disease burden compared with mice receiving control Th1 cells (Fig. 4B); however, mice receiving GSK3 inhibitor treated cells and anti-IL-10R Ab displayed an increase in disease burden compared with controls (Fig. 4B).

### GSK3 inhibition causes epigenetic changes in the IL-10 promoter in murine and human CD4^+^ T cells

**Figure 5:**
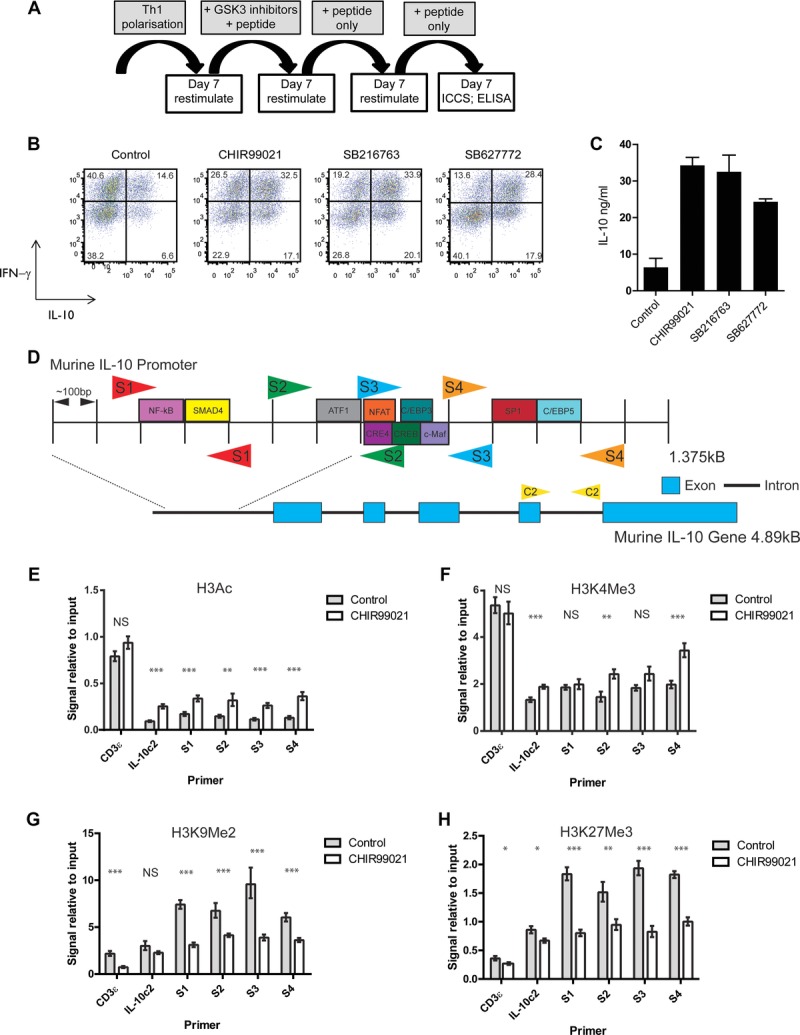
Stability of IL-10 expression and epigenetic analysis of the IL-10 promoter. (A) Schematic diagram showing experimental time-course. Th1 polarized Tg4 cells were restimulated with APCs in the presence of GSK3 inhibitors or vehicle control and MBP Ac1-9. IL-2 was added on day 3 of culture. On day 7 of restimulation, CD4^+^ T cells were restimulated with APCs in the presence of MBP Ac1-9 only (with no inhibitors). On day 7 CD4^+^ T cells were restimulated again with APCs only in the presence of MBP Ac1-9. (B) Intracellular cytokine staining for IFN-γ and IL-10 was carried out on day 7 of the final round of restimulation. Data are plots gated on live CD4^+^ cells and are representative of three independent experiments. (C) Tissue culture supernatants were taken on day 3 of the final round of stimulation and analyzed for IL-10 by ELISA. Data are mean + SEM of triplicate wells and are representative of two independent experiments. (D) Schematic diagram showing the positions of primer sets used in murine ChIP experiments. Four sets of primers spanning the IL-10 promoter (S1–S4) were used together with nonpromoter control primers (C2). Consensus transcription factor binding sites are shown. (E–H) Tg4 splenocytes were cultured in the presence of MBP Ac1-9 under Th1 polarizing conditions. On day 7 of culture, Th1 cells were restimulated with APCs in the presence of CHIR99021 or vehicle control as well as MBP Ac1-9 for further 7 days. IL-2 was added on day 3 of culture. Live CD4^+^ T cells were selected and ChIP analysis carried out using antibodies against acetyl histone H3 (E), H3K4me3 (F), H3K9me2 (G), and H3K27me3 (H). Graphs show the mean amount of immunoprecipitated DNA compared to the total amount of DNA, ± SEM, for three independent experiments. NS, not significant; *p < 0.05, **p < 0.01, ***p < 0.001 (*t*-test).

IL-10-mediated suppression of EAE in mice receiving GSK3 inhibitor treated Th1 cells by adoptive transfer was sustained throughout the 20-day course of the experiment, suggesting that the cellular changes effected by the inhibitor were long lasting. Indeed, Th1 cells cultured with inhibitor expressed higher levels of IL-10 than controls for several rounds of restimulation after inhibitor removal (Fig. 5A–C). This led us to investigate the epigenetic status of the IL-10 promoter since epigenetic changes are able to influence prolonged changes in gene expression. ChIP analysis of the murine IL-10 promoter, using four primer sets spanning the promoter (Fig. 5D), revealed a significant GSK3 inhibitor induced increase in histone H3 acetylation, a marker for active gene transcription (Fig. 5E). There was also a modest increase in histone H3 trimethylation (H3K4me3, Fig. 5F). Strikingly, however, there were dramatic decreases in two repressive histone marks, histone H3 lysine 9 dimethylation (H3K9me2) and histone H3 lysine 27 trimethylation (H3K27me3) following GSK3 inhibitor treatment of Th1 cells (Fig. 5G and H).

**Figure 6:**
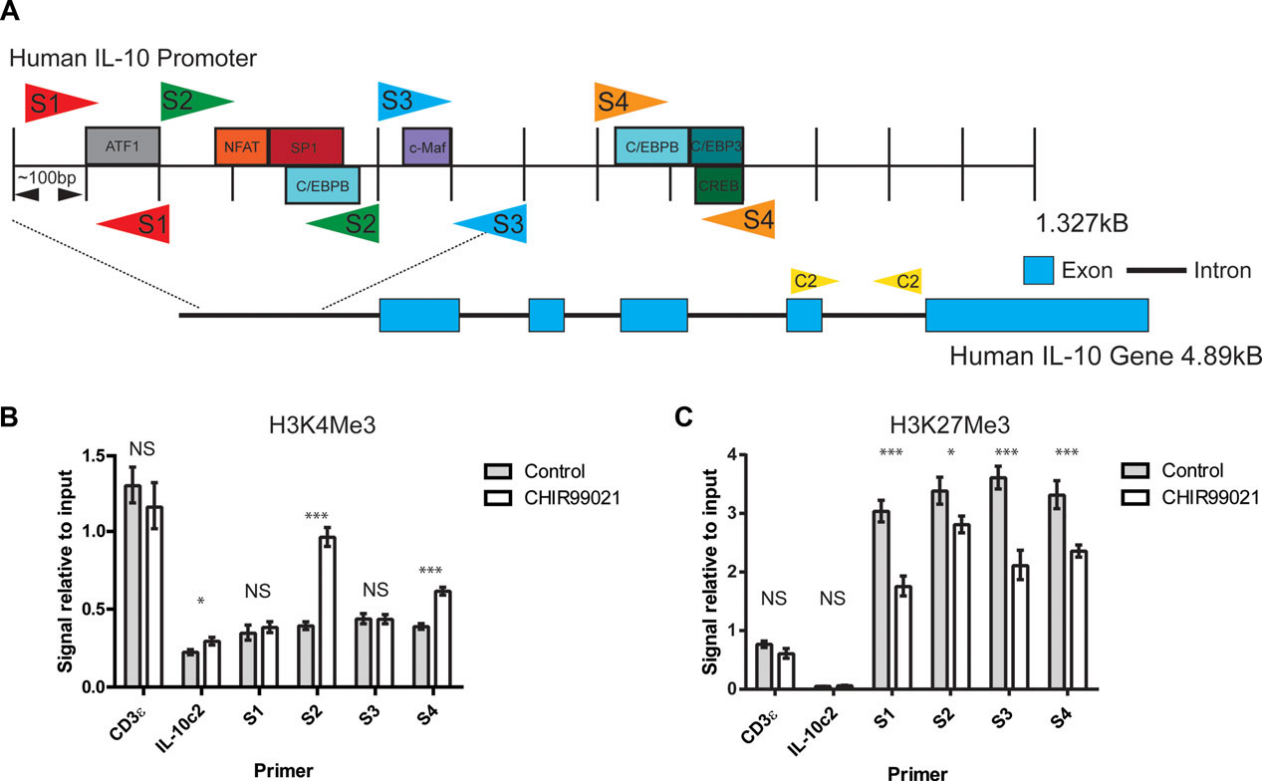
Epigenetic analysis of the IL-10 promoter in human memory T cells. (A) Schematic diagram showing the positions of primer sets used in human ChIP experiments. Four sets of primers spanning the IL-10 promoter (S1-S4) were used together with nonpromoter control primers (C2). Consensus transcription factor binding sites are shown. Memory CD4^+^ T cells were isolated from PBMC by magnetic selection and stimulated with anti-CD3 and anti-CD28 in the presence of IL-2 and CHIR99021 and vehicle control for 7 days. Live CD4^+^ T cells were selected and ChIP analysis carried out using antibodies against H3K4me3 (B) and H3K27me3 (C). Graphs show the mean amount of immunoprecipitated DNA compared to the total amount of DNA, ± SEM, for four independent experiments. NS, not significant; *p < 0.05, **p < 0.01, ***p < 0.001 (*t*-test).

We also investigated whether similar epigenetic modifications occurred in human CD4^+^ effector T cells. Memory CD4^+^ T cells were analyzed for the presence of H3K4me3 and H3K27me3, in the IL-10 promoter after culture with GSK3 inhibitor (Fig. 6A–C). Changes in these chromatin modifications were remarkably similar to those observed in murine Th1 cells with a significant upregulation of H3K4me3 at the regions covered by primer sets S2 and S4 (Fig. 6B) and a decrease in H3K27me3 across the IL-10 promoter (Fig. 6C).

### GSK3 inhibitor treatment of Th1 cells causes the upregulation of Nfil3, c-Maf, and GATA3

**Figure 7:**
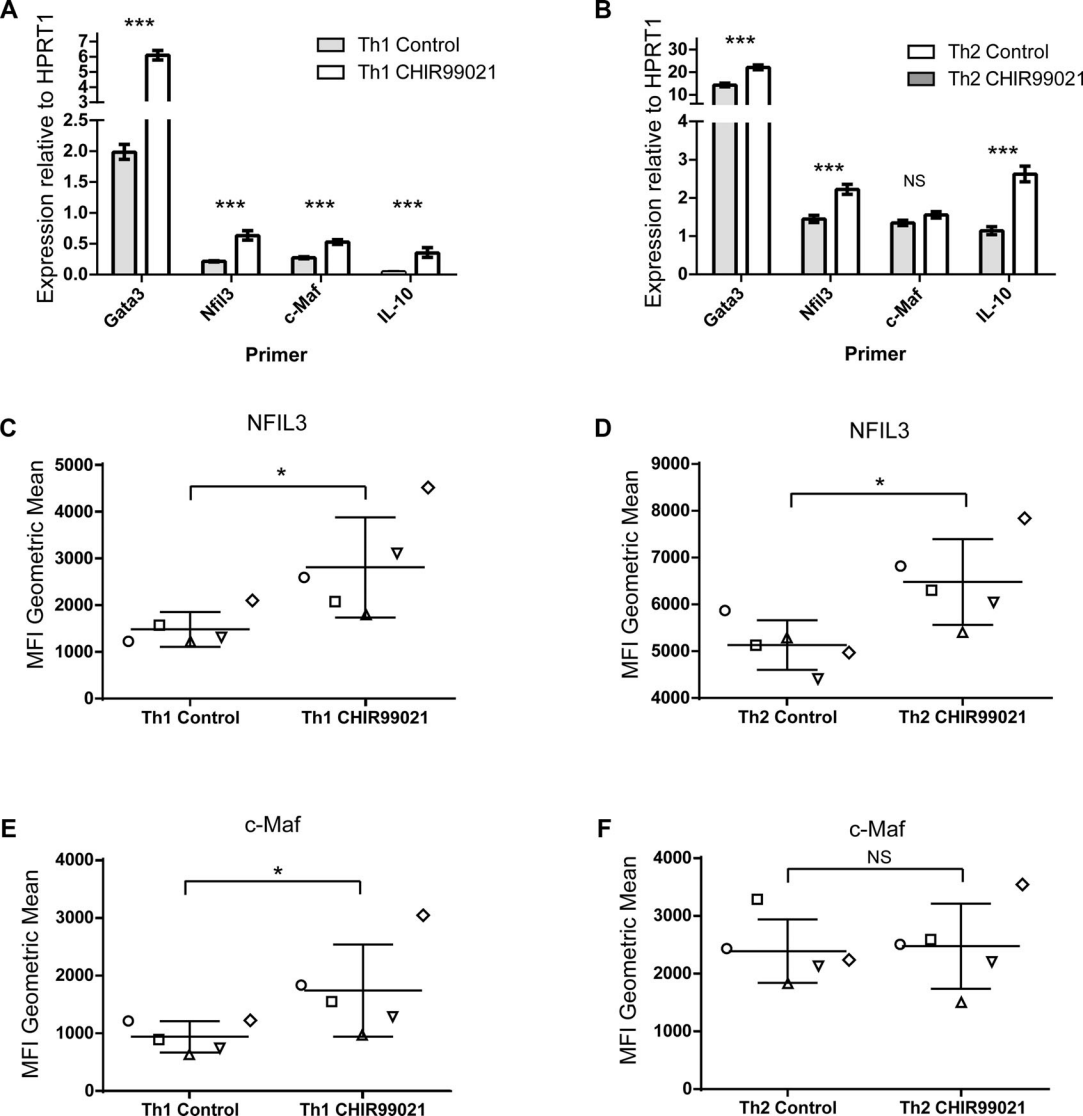
Transcription factor expression changes induced by GSK3 inhibition. Tg4 splenocytes were cultured in the presence of peptide MBP Ac1-9 (10 μg/mL) under Th1 or Th2 polarizing conditions. On day 7 of culture, Th1 and Th2 cells were restimulated with APCs in the presence of CHIR99021 or vehicle control as well as peptide MBP Ac1-9 (10 μg/mL) for further 7 days. IL-2 was added on day 3 of culture. (A and B) mRNA was purified from live CD4^+^ T cells for RT-PCR analysis. Graphs show mRNA expression relative to the housekeeping gene hypoxanthine phosphoribosyltransferase 1 (HPRT1) for five pooled experiments. NS, not significant; ***p < 0.001 (*t*-test). (C–F) Cells were stimulated with PMA and ionomycin and intracellular transcription factor staining was carried out. Results were gated on live CD4^+^ cells and the geometric mean florescence of transcription factor staining was calculated using FlowJo software. Graphs show mean ± SEM. A paired two-tail *t*-test analysis was carried out on pooled data from five independent experiments. NS, not significant; *p < 0.05.

GSK3 is known to influence several transcription factors (reviewed in 10,11). We, therefore, examined the expression of three transcription factors, Nfil3, c-Maf, and GATA3, previously shown to affect IL-10 production in CD4^+^ T cells. Analysis by mRNA quantitation and protein expression in murine cells showed an increase in each of these transcription factors in Th1 cells upon GSK3 inhibition (Fig.7A, C, and E). Levels of all three transcription factors were found to be higher in Th2 cells than in Th1 cells. GATA3 and Nfil3 expression was further increased in Th2 cells upon GSK3 inhibition, however, no change was observed in c-Maf in Th2 cells (Fig.7B, D, and F). Both GATA3 and c-Maf are known to be important transcription factors in Th2 cells 28,29, however, no upregulation of IL-4 upon GSK3 inhibition was observed in either Th1 or Th17 cells (Supporting Information Fig. 9) and, additionally, no difference in phospho-STAT6 levels in Th1 or Th2 cells cultured with GSK3 inhibitor (Supporting Information Fig. 10)

## Discussion

We have extended previous studies by analyzing the effect of GSK3 inhibition on CD4^+^ effector T-cell subsets; Th1, Th2, and Th17 lineages all responded to GSK3 inhibitors with an increase in secreted IL-10 protein. Adoptive transfer of GSK3 inhibitor treated Th1 cells demonstrated the protective effect of IL-10 secreted by these cells. Lithium treatment was previously shown to decrease severity of EAE whether treatment was administered prior to or after disease onset 17. Lithium was found to decrease peptide-induced IL-6, IL-17, and IFN-γ from T cells; the authors suggested the effect on EAE could be due to a reduction in Th17 cells. A subsequent study showed that GSK3 inhibition blocked the differentiation of Th17 cells 19. In a further model of Th1-induced EAE, lithium was shown to induce IL-27 in the CNS of mice 30 with a trend toward increased IL-10. We have shown that GSK3 inhibition is able to directly induce IL-10 production in CD4^+^ T cells without concomitant production of IL-27 by APCs (data not shown); however, it is possible that IL-27 production may contribute to IL-10 production in vivo. Another study found that GSK3 inhibition led to reduced differentiation of Th1 cells and decreased severity of Th1-mediated EAE 18. If GSK3 inhibitors were administered after the onset of disease then amelioration of symptoms was also observed 18. In our study, Th1 cells were used to induce disease and the protective effect of GSK3 inhibition was principally through the induction of IL-10 secretion. Therefore, GSK3 inhibitors may be therapeutic in either Th1- or Th17-driven conditions since GSK3 inhibition also ameliorates Th17-related pathology through IL-10-independent mechanisms.

Th2 differentiated cells upregulated IL-10 upon GSK3 inhibitor treatment and this may be important in the context of allergen-specific immunotherapy. Ag-specific immunotherapy in allergy to house dust mite, birch pollen, bee venom, and grass pollen 31–33 is characterized by an increase in IL-10-producing T cells. IL-10 may have an important role in regulating the balance between IgE and IgG4 antibodies with associated counterinflammatory consequences 34. In the presence of IL-4, an increase in IL-10 leads to a preferential class switch in favor of IgG4 and both total and allergen-specific IgE responses are suppressed 35,36. This beneficial effect of a rise in IL-10 in the presence of IL-4, as seen in this study, suggests that GSK3 inhibition may be a useful tool in allergen-specific immunotherapy.

There are several levels of control of differentiation of CD4^+^ T cells. Transcription factors such as T-bet for Th1 cells and FoxP3 for Treg cells are crucial for the regulation of lineage-specific gene expression. However, epigenetic mechanisms also emerge as critical regulators of differentiation (reviewed in 37–39). PTMs of histones affect gene expression by altering DNA accessibility. Epigenetic changes may influence both differentiation and maintenance of cellular phenotype as the changes in chromatin structure are maintained in cells and inherited by their descendants 40. Several groups have previously found a correlation between epigenetic modifications and gene expression in CD4^+^ T cells 41–45.

Histone H3 acetylation and Lys-4 trimethylation (H3K4me3) permit greater accessibility of DNA to transcription factors, whereas Lys-27 trimethylation (H3K27me3) or Lys-9 dimethylation (H3K9me2) are associated with gene repression. While these methylation events on H3 have clear contrasting effects on gene expression, it is believed that genes marked with both H3K4me3 and H3K27me3 remain in a “poised” state ready for later activation 46. Although CD4^+^ T cells have been thought to be distinct lineages defined by the transcription factors they express and the cytokines they produce, there is in fact considerable plasticity in CD4^+^ T-cell fate 47; indeed, there are several possible combinations of activating and repressive modifications 48. This flexibility may allow the capacity for more adaptive responses in a changing environment or immunological challenge.

Here we have studied changes in H3 modification at the IL-10 promoter in response to inhibition of GSK3. In murine Th1 cells, GSK3 inhibition effects a change in the chromatin modifications associated with the IL-10 promoter from the repressive H3K9me2 and H3K27me3 to the activating H3Ac and H3K4me3 and these changes may allow the chronic maintenance of the IL-10-expressing phenotype. In human memory CD4^+^ T cells, we also observed an increase in H3K4me3 and a decrease in H3K27me3 at the IL-10 promoter upon GSK3 inhibition, strengthening our suggestion that GSK3 inhibition may be a means of upregulating IL-10 production in autoimmune or allergic disease.

Epigenetic changes in gene expression can be facilitated by environmental agents and so may be particularly important in the development of autoimmune disease, which is thought to occur in genetically susceptible individuals exposed to an environmental trigger 49–51. Therefore, the changes in cytokine production in CD4^+^ T cells elicited by GSK3 inhibition may also suggest a mechanism whereby dysregulation of GSK3 may render an individual susceptible to autoimmune disease; in fact, a particular allelic variant of GSK3β is a susceptibility factor for multiple sclerosis 52.

Epigenetic changes involve the physical remodeling of chromatin structure that in turn regulates accessibility to transcription factors. GSK3 is known to influence the activity of many transcription factors and regulate transcription of cytokines in several other systems studied 10,53. Nfil3 is known to influence IL-10 expression in both Th1 and Th2 cells 54–56. c-Maf is essential for IL-10 expression in macrophages 57 and its expression correlates with IL-10 expression in CD4^+^ T cells 58–60. GATA3 induces the differentiation of Th2 cells and inhibits that of Th1 cells 28,61. Our observations show that Nfil3 and GATA3 are upregulated upon GSK3 inhibition in Th1 and Th2 cells and c-Maf is significantly upregulated in Th1 cells only. c-Maf is highly expressed in Th2 cells and so it is possible that further upregulation is not possible or that the c-Maf locus in Th2 cells is modified in a manner that precludes further upregulation by GSK3 inhibition. Interestingly, GATA3 has also been shown to induce changes in the chromatin structure at the IL-10 locus in the absence and presence of IL-4 62 and Nfil3 has also been reported to influence histone modifications 63. Therefore, the increase in GATA3 and Nfil3 expression upon GSK3 inhibition could be contributing to the changes in histone modifications observed in this study. These findings elucidate a novel regulation of these transcription factors by GSK3 and their upregulation correlating with the increase in IL-10. We conclude that GSK3 inhibition leads to a stable upregulation of IL-10 in Th1 and Th2 cells, which may be beneficial in the development of new therapies for autoimmune and allergic diseases.

## Materials and methods

Experimental mice were maintained under specific pathogen-free conditions. B10.PL (*H2u*) mice were obtained from The Jackson Laboratory. The Tg4 TCR transgenic mouse expressing Vβ8.2 TCR specific for MBP Ac1-9 and the B10.PL *IL-10*^−/−^ mouse have been described previously 6. All experiments were approved by the UK Home Office and performed according to animal welfare codes directed by the University of Bristol ethical review committee.

### Murine cell separation

Purified CD4^+^ T cells were isolated by magnetic separation using the CD4^+^ T cell Isolation kit II (Miltenyi Biotec) or the EasySep Mouse Naive CD4^+^ T cell Isolation Kit (Stemcell Technologies).

### Murine cell culture

Splenocytes were cultured in RPMI medium (Lonza) containing heat-inactivated FCS (5%, BioSera), HEPES buffer (Lonza), β-mercaptoethanol (Gibco), glutamine and penicillin/streptomycin (both Lonza) as well as 10 μg/mL peptide Ac1-9 of MBP (AcASQKRPSQR; GL Biochem, Shanghai). For Th1 polarization rmIL-12 (5 ng/mL; Peprotech) and anti-IL-4 (11B11; 10 μg/mL, BioXCell) were added and for Th2 polarization rmIL-4 (10 ng/mL, Peprotech) and anti-IFN-γ (XMG1.2) (10 μg/mL, BioXCell) were added. For Th17 polarization rmIL-6 (25 ng/mL, Peprotech), rmIL1β (10ng/mL, Peprotech), rhTGF-β1 (2ng/mL, Peprotech), anti-IFN-γ (XMG1.2, 25 μg/mL), and anti-IL-4 (11B11, 10 μg/mL) were added. rhIL-2 (R&D Systems) was added at 20 U/mL on day 3 for both Th1 and Th2 cultures and rhIL-23 (Peprotech) at 5 ng/mL for Th17 cultures. GSK3 inhibitors were used at the following predetermined optimal concentrations: CHIR99021 (2 μM, Tocris), SB216763 (5 μM, Tocris), SB627772 (10 μM, supplied by GlaxoSmithKline (Stevenage) or synthesized by Avistron Chemistry Services), L803mts (100 μM, Tocris). On day 7, live CD4^+^ T cells were isolated by Ficoll centrifugation and restimulated with irradiated B10.PL splenocytes. Alternatively, live CD4^+^ T cells were isolated by Ficoll centrifugation and then magnetic separation using the CD4^+^ T cell Isolation kit II and restimulated with either anti-CD3 and anti-CD28 coated beads (Dynabeads, Life Technologies) or on anti-CD3 (1 μg/mL, eBioscience; clone 145-2C11) and anti-CD28 (2 μg/mL, eBioscience; clone 37.51) coated plates.

### Intracellular cytokine and transcription factor staining

Cells were stimulated with medium containing PMA (5ng/mL), ionomycin (500 ng/mL), and GolgiStop (BD Biosciences) for 3 h at 37°C. Cells were stained with fixable viability dye ef780 (eBioscience) and then surface-stained with anti-CD4 Alexa700. Following incubation with IC fixation buffer (eBioscience), cells were stained intracellularly with anti-IFN-γ PECy7, anti-IL-10 allophycocyanin, anti-IL-17A PE, and/ or anti-IL-4 PE (all eBioscience). For intracellular transcription factor staining, cells were fixed by incubation with the FoxP3 Staining Buffer Set (eBioscience). The antibodies anti-CD4 FITC, anti-Nfil3 PE, and anti-c-Maf ef660 and their corresponding isotype controls (all eBioscience) were used. Fluorescence intensity was measured on a BD LSR II flow cytometer and analyzed using FlowJo (Tree Star Inc.) software. FACS gating strategy is shown in Supporting Information Method 1.

### Cytokine protein levels

IL-10 levels in tissue culture supernatant were measured using a FlowCytomix fluorescent bead immunoassay (eBioscience) according to manufacturer's instructions. Fluorescence intensity was measured on a BD Calibur flow cytometer and analyzed using FlowCytomix Pro software (eBioscience). IL-10 protein levels were also measured using ELISA with paired antibodies according to the manufacturer's instructions (Peprotech). IFN-γ protein levels were measured using ELISA with paired antibodies (BD Biosciences). Optical densities were read at 450/595 nm on a Spectramax190 microplate reader and analyzed using SoftMax Pro software (Molecular Devices).

### Isolation of human T-cell subsets

CD leukocyte cones from healthy volunteers were obtained from the National Health Service Blood and Transplant. Ethical approval was granted by NHS-research ethical committee (NHS-REC 13/NW/0275). PBMCs were isolated by Ficoll separation. Magnetic-based techniques were then used to isolate naive, memory, and CD4^+^ T cells (Naive CD4^+^ T cell isolation kit II, Memory CD4^+^ T cell isolation kit and CD4^+^ T cell isolation kit II, respectively, all from Miltenyi Biotec). Naive or memory cell purity was greater than 95%, based on expression of CD45RA or CD45RO, respectively. For flow cytometric sorting of effector cell subsets, pre-enriched CD4^+^ T cells were stained for CD4, CXCR3, CCR4, and CCR6, with 7-AAD, and sorted three ways using a BD Influx cell sorter (BD Biosciences); Th1 cells (viable CD4^+^CXCR3^+^), Th2 cells (viable CD4^+^CXCR3^−^CCR4^+^CCR6^−^), or Th17 cells (viable CD4^+^CXCR3^−^CCR4^+^CCR6^+^).

### Culture of human T-cell subsets

Naive and memory human CD4^+^ T cells were cultured on anti-CD3 (clone OKT3, 10 μg/mL; BioXCell) and anti-CD28 (clone 9.3, 2 μg/mL; BioXCell) coated plates with 20 U/mL IL-2 in RPMI 1640 (Lonza), supplemented with 10% heat-inactivated FCS (BioSera), penicillin/streptomycin and 2 mM L-glutamine (both Lonza), 2-mercapto-ethanol (Gibco), HEPES buffer (Lonza), 100 mM sodium pyruvate (Gibco), and and 1% nonessential amino acids (Sigma). Cells were split and media replenished as necessary.

Th1, Th2, and Th17 cells were cultured with autologous irradiated APCs (CD4^−^ cells) at a 1:5 ratio, with 100 U/mL IL-2 and 1 μg/mL anti-CD3 (OKT3) in X-VIVO 15 media (Lonza), supplemented with 5% human AB serum (Sigma), penicillin/streptomycin, and L-glutamine. GSK-3 inhibitors were added as above and cells were split as necessary. To determine cytokine production, on day 7 of T-cell culture, cells were stimulated with PMA (50 ng/mL) and ionomycin (1 μg/mL) for 4 h, in the presence of Golgi Stop. Cells were stained with fixable viability dye ef780 (eBioscience) and surface-stained with anti-CD4 Alexa700. Following fixation and permeabilization, intracellular staining for anti-IL-10 ef660, anti-IFNγ PerCPCy5.5, anti-IL-17A PE, and anti-IL-4 FITC (all eBioscience) was carried out.

### EAE

EAE was induced in Tg4 mice on day 0 by i.p. injection of 10^7^ Th1 cells. Clinical signs of EAE were assessed daily with a scoring system as follows: 0, no disease; 1, flaccid tail; 2, impaired righting reflex and/ or partial hind-leg paralysis; 3, total hindlimb paralysis; 4, fore- and hindlimb paralysis; 5, moribund or dead.

### ChIP analysis

ChIP was carried out using the EZ-Magna ChIP A Chromatin Immunoprecipitation Kit (Millipore). A total of 20 × 10^6^ CD4^+^ T cells were fixed in 1% formaldehyde (Fisher Scientific). Glycine buffer was added to quench any remaining formaldehyde. Cells were then lysed and sonicated using a Diagenode bath sonicator at high setting with 30-s pulses over a 5-min period. Immunoprecipitation was carried out using the following antibodies: anti-acetyl-histone H3 (Millipore), ChIPAb+ Trimethyl-Histone H3(Lys4) (Millipore), ChIPAb+ Trimethyl-Histone H3(Lys27) (Millipore), anti-Histone H3 (di methyl K9) (Abcam), and rabbit IgG (Millipore). Real-time PCR analysis was carried out using the MJ Opticon 2 thermo cycler (MJ Research) and Platinum SYBR Green qPCR SuperMix-UDG (Invitrogen). Cycling conditions were set up according to the manufacturer's instructions; annealing temperature for all primers was 55°C with 40 cycles of amplification. For ChIP primers and statistical analysis, see Supporting Information Method 2.

### mRNA analysis

Total mRNA from 3–5 × 10^6^ CD4^+^ T cells after overnight anti-CD3 and anti-CD28 stimulation was isolated using RNAeasy Mini Kit (Qiagen) and quality and quantity were verified using a Nanodrop (Thermo scientific). cDNA synthesis was carried out using SuperScript III First-strand Synthesis SuperMix for qRT-PCR (Invitrogen). Real-time PCR was carried out using the MJ Opticon 2 thermo cycler (MJ Research) and Taqman probes (GATA3, NFIL3, c-Maf, IL-10, HPRT1; Life Technologies). Expression of the target gene was obtained by using the following equation: target gene expression = 2^(mean HPRT1 ct − mean target gene ct)^.

## Abbreviations

EAE: experimental autoimmune encephalomyelitis
GSK3: glycogen synthase kinase-3
MBP: myelin basic protein

## Supporting Information

As a service to our authors and readers, this journal provides supporting information supplied by the authors. Such materials are peer reviewed and may be re-organized for online delivery, but are not copy-edited or typeset. Technical support issues arising from supporting information (other than missing files) should be addressed to the authors.

Figure s1 Figure s2 Figure s3 Figure s4 Figure s5 Figure s6 Figure s7 Figure s8 Figure s9 Figure s10
