# Supplementary material for: Glycogen synthase kinase-3 controls IL-10 expression in CD4+ effector T-cell subsets through epigenetic modification of the IL-10 promoter
Source: Eur J Immunol. 2015 Feb 17;45(4):1103–15. doi: 10.1002/eji.201444661 (PMC4405077; doi:10.1002/eji.201444661)
Supplement: Supplementary file 1 — Figure s1 Figure s2 Figure s3 Figure s4 Figure s5 Figure s6 Figure s7 Figure s8 Figure s9 Figure s10 [file eji0045-1103-sd1.pdf]

Supporting Information Methods 1

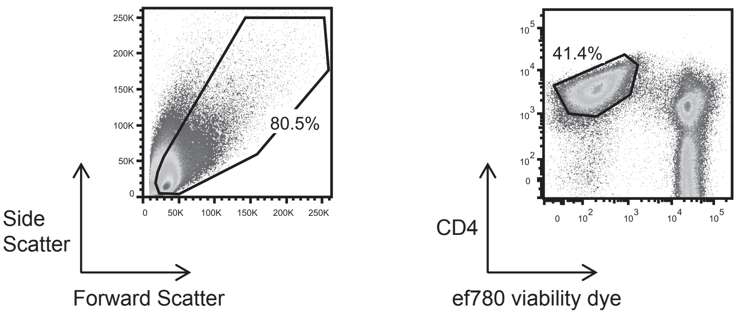

FACS gating strategy.

An example is shown of the FACS gating strategy used. Lymphocytes were gated in a FSC/ SSC plot as indicated and live CD4$^+$ cells within this population were gated in a CD4-Alexa700 / ef780 viability dye plot as indicated. The live CD4$^+$ cells were then further analysed for intracellular cytokines or transcription factors.

Supporting Information Methods 2

Primers for murine ChIP:

Primer sequences:

IL-10 C2: Forward AATGGTGTGACCTCCTCTGC ; Reverse AATGGTGTGACCTCCTCTGC. CD3ε: Forward CTGCCTCAAACATTTCCAAGTGA; Reverse ACACAGTGGCTGCATGCAAAG. IL-10 Promoter S1: Forward GTCAACAGGACGTGTAGCAT; Reverse GGAGACGTGTAACCTGTAGC. IL-10 Promoter S2: Forward CTGGGATCTGAGCTTCTTCG; Reverse GACTCGGAAGTCACCTTAGC. IL-10 Promoter S3: Forward GAGTGCTAAGGTGACTTCCGA; Reverse AACTTCTGCATTACAGCTATTTTT. IL-10 Promoter S4: Forward GCAGAAGTTCATTCCGACCA; Reverse GGCATGATGGAGCTCTCTTT

Primers for human ChIP:

IL-10 C2: Forward TCTGTTAAGTGTCTTTGGGG ; Reverse TGAACCAACTCCAGGTTAAG. CD3ε: Forward TTCCAAGTGAGGTAAAACCC; Reverse TCTGCTAGAGATAAGGACCC. IL-10 Promoter S1: Forward TGAAGAAGTCCTGATGTCAC; Reverse TTACCTATCCCTACTTCCCC. IL-10 Promoter S2: Forward GGGGAAGTAGGGATAGGTAA; Reverse CAGTGCCAACTGAGAATTTG. IL-10 Promoter S3: Forward AGCACTACCTGACTAGCATA; Reverse AGAGACTGGCTTCCTACAG. IL-10 Promoter S4: Forward GGGGACCCAATTATTTCTCA; Reverse TGGGCTACCTCTCTTAGAAT

Statistical methods for ChIP and mRNA experiments:

Median fold change compared to input DNA was obtained by using the following equation; Fold change compared to input DNA = $2^{(\text{mean input DNA ct- mean sample DNA ct})}$.

The Sums of squares (SS) and Degrees of freedom (DF) for control and treatment group is obtained from a 2-way ANOVA analysis performed on the Ct values in Graphpad Prism. The variance is obtained by $Variance = {(SS_1 + SS_2)}/{(DF_1 + DF_2)}$. The standard error is obtained by $Standard\ Error = \sqrt{{1}/{n_{a1}} + {1}/{n_{b1}} + {1}/{n_{a2}} + {1}/{n_{b2}}}$. The subscript numbers represents the control/treatment group, the “a” represents the input DNA or housekeeping gene whilst the “b” represents the target gene or immunoprecipitated DNA, “n” represents the number of repeats in each group (including technical and biological repeats). T is then determined by $t = {[|\Delta\overline{Ct_1} - \Delta\overline{Ct_2}|]}/{Standard\ Error}$ the p value is then determined by referring to a T test table for t and $(DF_1 + DF_2)$ degrees of freedom.

Supporting Information Figure 1

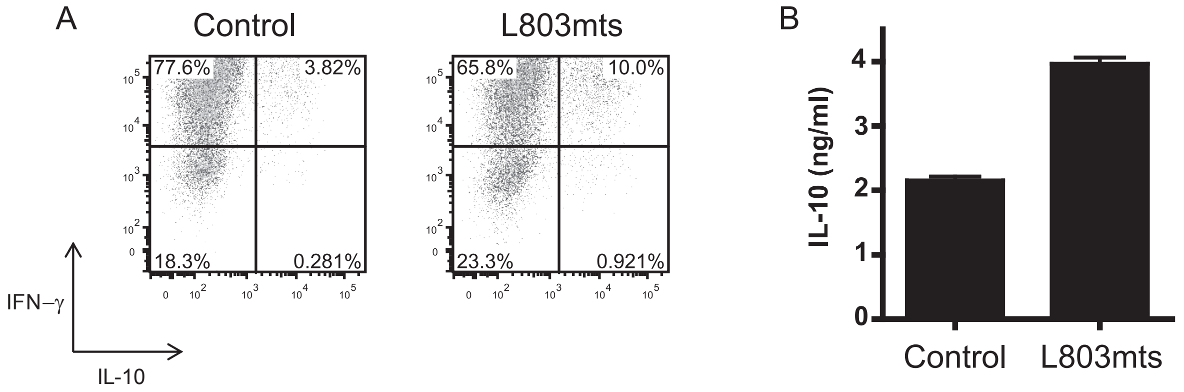

The substrate - competitive GSK3 inhibitor L803mts increases IL-10 expression in Th1 cells.

A. Tg4 splenocytes were cultured in the presence of peptide MBP Ac1-9 (10μg/ml) under Th1 polarising conditions. On day 7 of culture, Th1 cells were restimulated with APCs in the presence of L803mts (100μM) or vehicle control as well as peptide MBP Ac1-9 (10μg/ml). IL-2 was added on day 3 of culture and intracellular cytokine staining for IFN-γ and IL-10 carried out on day 7 of stimulation. Data are plots gated on live $CD4^+$ cells and are representative of two independent experiments.

B. Supernatants were taken from cultures on day 7 of restimulation and analysed for the presence of IL-10 by ELISA. Data are the mean and SEM of triplicates from one of two independent experiments.

Supporting Information Figure 2

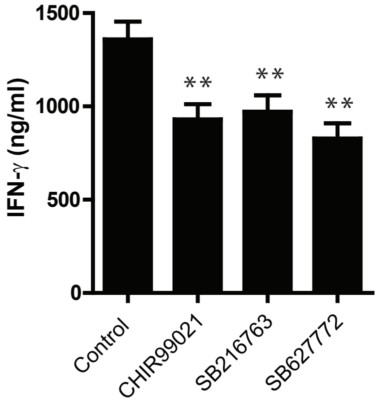

GSK3 inhibition leads to a decrease in secreted IFN-γ from Th1 cells.

Tg4 splenocytes were cultured in the presence of peptide MBP Ac1-9 (10μg/ml) under Th1 polarising conditions. On day 7 of culture, Th1 $CD4^+$ T cells were isolated and restimulated with anti-CD3 and anti-CD28 in the presence of IL-2 and GSK3 inhibitors or vehicle control. Tissue culture supernatants were taken on day 7 of stimulation and analysed for IFN-γ by ELISA. Data are mean + SEM of three independent experiments carried out in triplicate. ** $p<0.01$ ANOVA with Dunnets Multiple Comparison post-test.

Supporting Information Figure 3

A

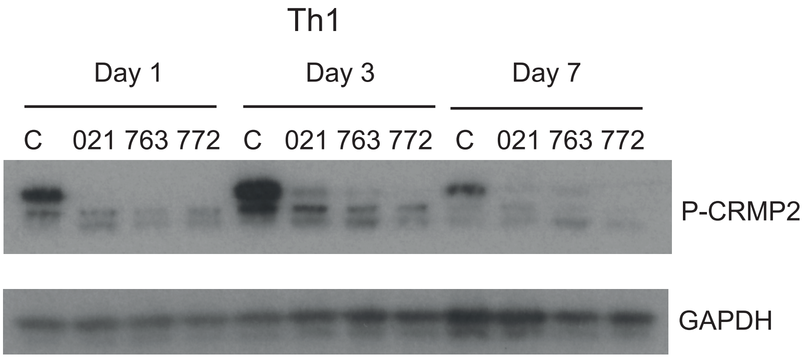

B

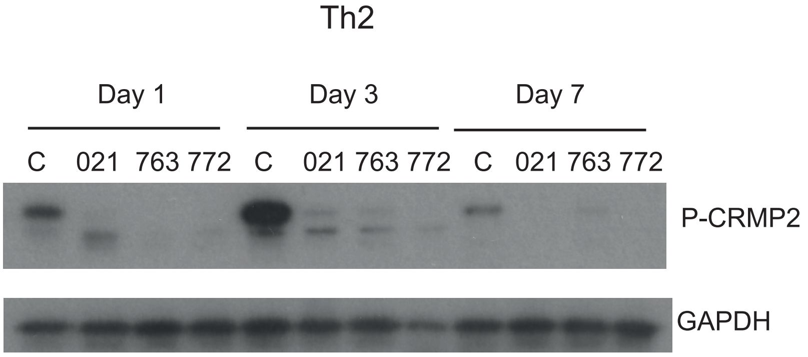

Analysis of GSK3 inhibtion by Western blotting for P-CRMP2.

Th1 (A) and Th2(B) polarised Tg4 CD4+ cells were cultured on anti CD3/ anti CD28 coated plates in the presence of the GSK3 inhibitors CHIR99021 (021), SB216763 (763), SB627772 (772) or vehicle control. On day 2 cells were transferred to an uncoated plate and on day 3 IL-2 added to the cultures.
Cells were removed from the cultures on day 1, day 3 and day 7 of stimulation, washed in PBS and lysed in RIPA buffer. Protein concentration was measured using BCA assay and equal amounts of protein run in each lane on a Western blot which was probed using anti-pCRMP2(T514) antibody (Abcam) and then goat anti-rabbit HRP secondary antibody. After developing, blots were stripped with glycine buffer and reprobed with anti-GAPDH antibody (Cell Signaling).
Blots shown are representative of 3 (Th1) or 2 (Th2) independent experiments.

Supporting Information Figure 4

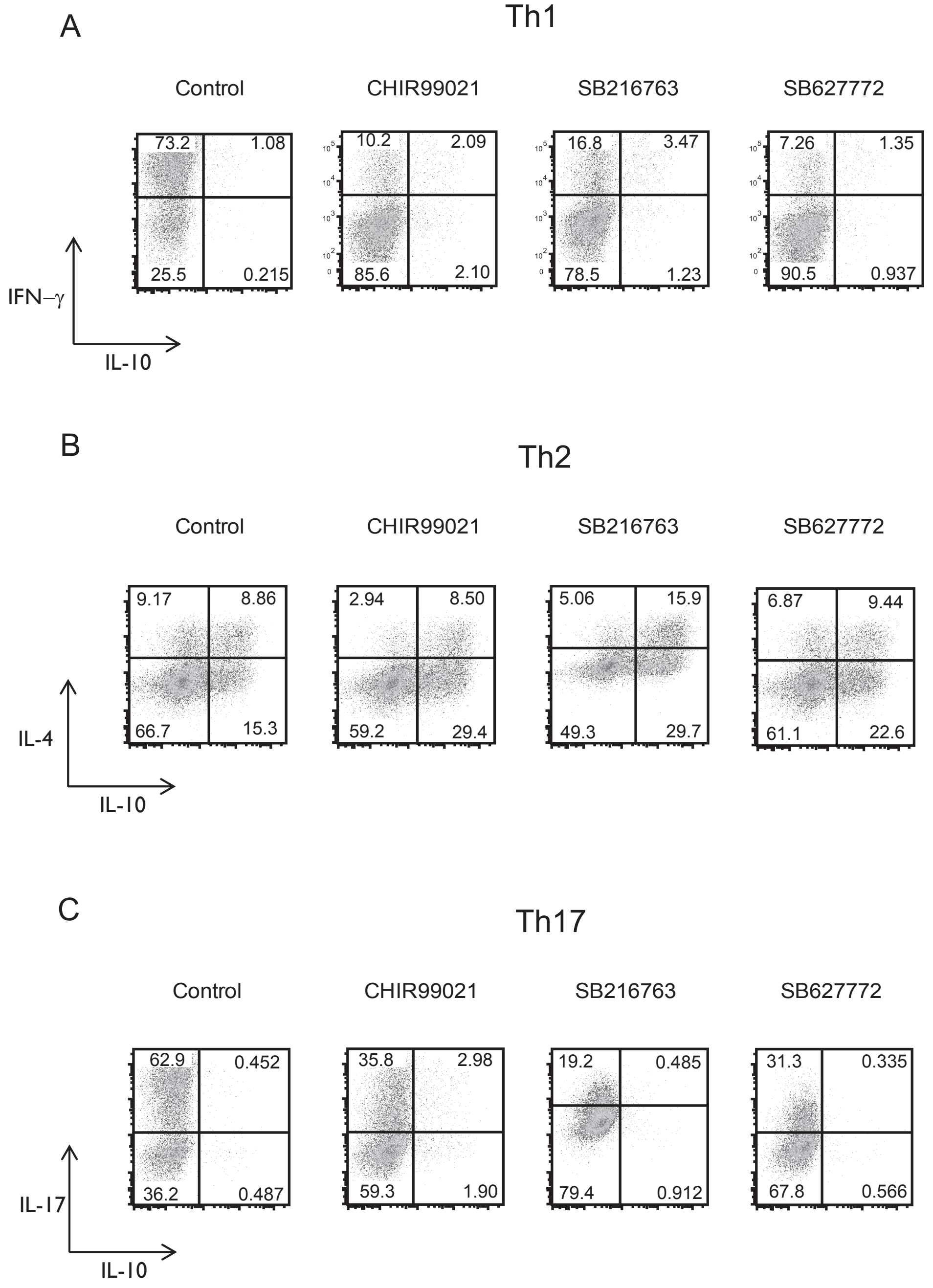

Effects of GSK3 inhibition on polarisation of Th1, Th2 and Th17.

Tg4 splenocytes were cultured in the presence of MBP Ac1-9 peptide and either for Th1 polarisation (A) rmIL-12 (5ng/ml) and anti-IL-4 (10μg/ml) were added or for Th2 polarisation (B) rmIL-4 (10ng/ml) and anti-IFN-γ (10μg/ml) were added. For Th17 polarisation (C) rmIL-6 (25ng/ml), rmIL1β (10ng/ml), rhTGF-β1 (2ng/ml), anti-IFN-γ (25μg/ml) and anti-IL-4 (10μg/ml) were added.
rhIL-2 was added at 20U/ml on day 3 for both Th1 and Th2 cultures and rhIL-23 at 5ng/ml for Th17 cultures.

On day 7 intracellular cytokine staining was carried out. Data are plots gated on live, $CD4^+$ cells and are representative of 3 independent experiments.

Supporting Information Figure 5

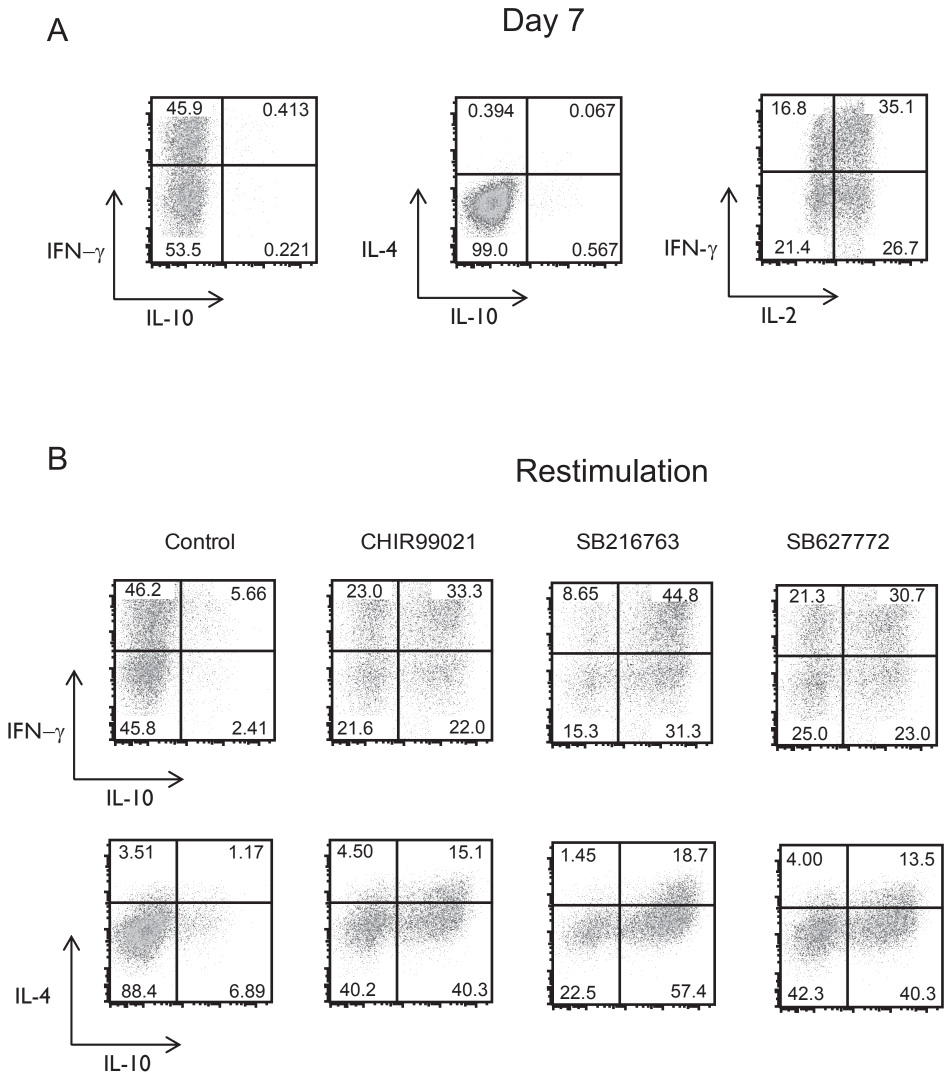

GSK3 inhibition induces IL-10 expression in restimulated cultures of unpolarised $CD4^+$ cells.

Splenocytes from a naive Tg4 mouse were cultured in the presence of MBP Ac1-9 peptide (10μg/ml) with no polarising cytokines or antibodies and on day 7 intracellular cytokine staining was carried out (A). Live $CD4^+$ cells were restimulated with APCs in the presence of MBP Ac1-9 peptide (10μg/ml), GSK3 inhibitors or vehicle control and intracellular staining was carried out on day 7 (B).
Data are plots gated on live, $CD4^+$ cells and are representative of 3 independent experiments.

Supporting Information Figure 6

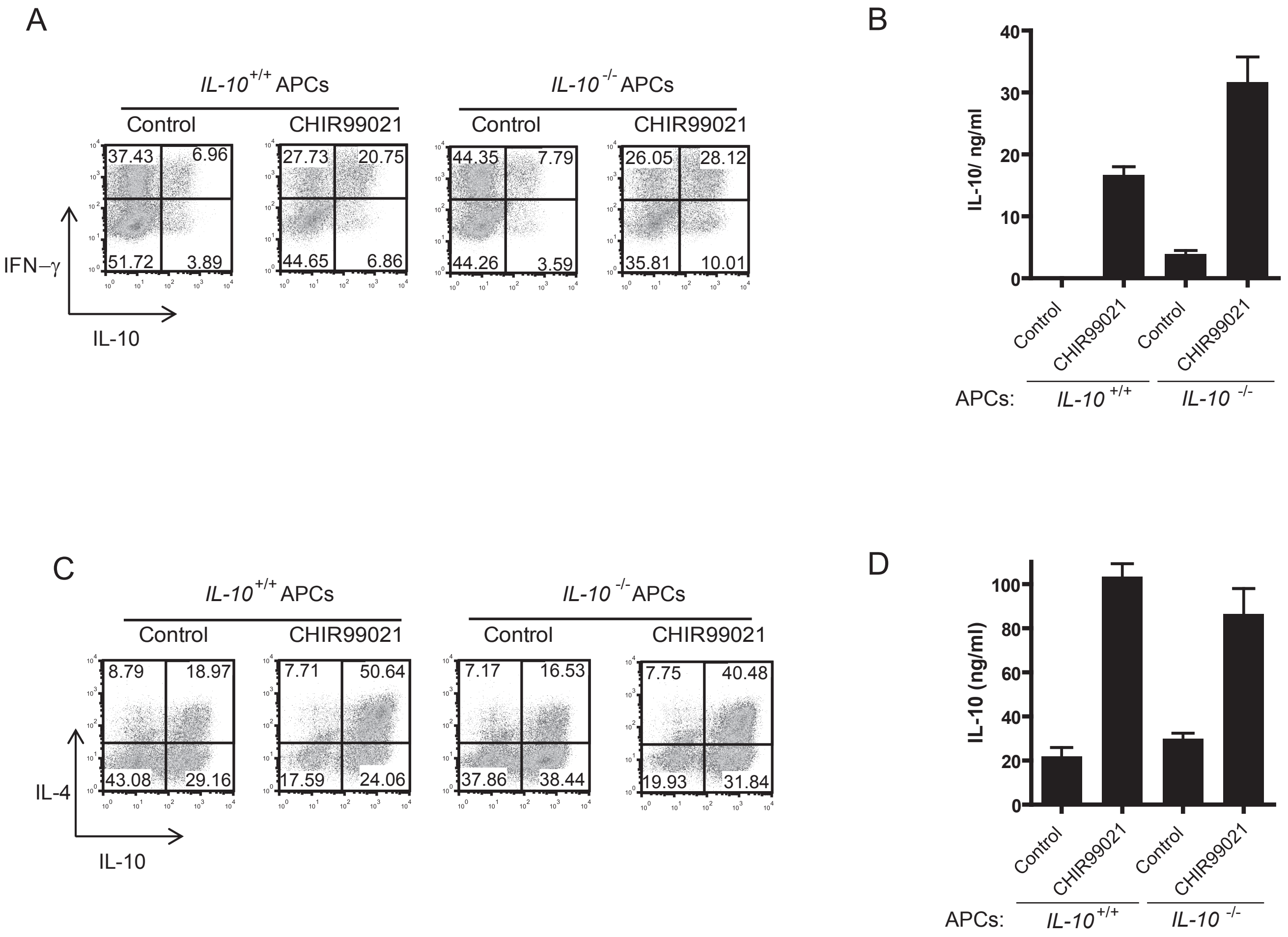

Exogenous IL-10 is not required for GSK3 inhibitor induced IL-10 production.

A. Tg4 splenocytes were cultured in the presence of peptide MBP Ac1-9 (10μg/ml) under Th1 polarising conditions. On day 7 of culture, Th1 cells were restimulated with APCs from either a B10.PL or B10.PL IL10-/- mouse in the presence of CHIR99021 or vehicle control. IL-2 was added on day 3 of culture and intracellular cytokine staining for IFN-γ and IL-10 carried out on day 7. Data are plots gated on live $CD4^+$ cells and are representative of 3 independent experiments.
B. Tissue culture supernatants were taken on day 7 of stimulation and analysed for IL-10 by ELISA. Data are mean + SEM of triplicate wells and are representative of 3 independent experiments.
C. Tg4 splenocytes were cultured in the presence of peptide MBP Ac1-9 (10μg/ml) under Th2 polarising conditions. On day 7 of culture, Th2 cells were restimulated with APCs from either a B10.PL or B10.PL IL10-/- mouse in the presence of CHIR99021 or vehicle control. IL-2 was added on day 3 of culture and intracellular cytokine staining for IL-4 and IL-10 carried out on day 7 of stimulation. Data are plots gated on live $CD4^+$ cells and are representative of 2 independent experiments.
D. Tissue culture supernatants were taken on day 7 of stimulation and analysed for IL-10 by ELISA. Data are mean + SEM of triplicate wells and are representative of 2 independent experiments

Supporting Information Figure 7

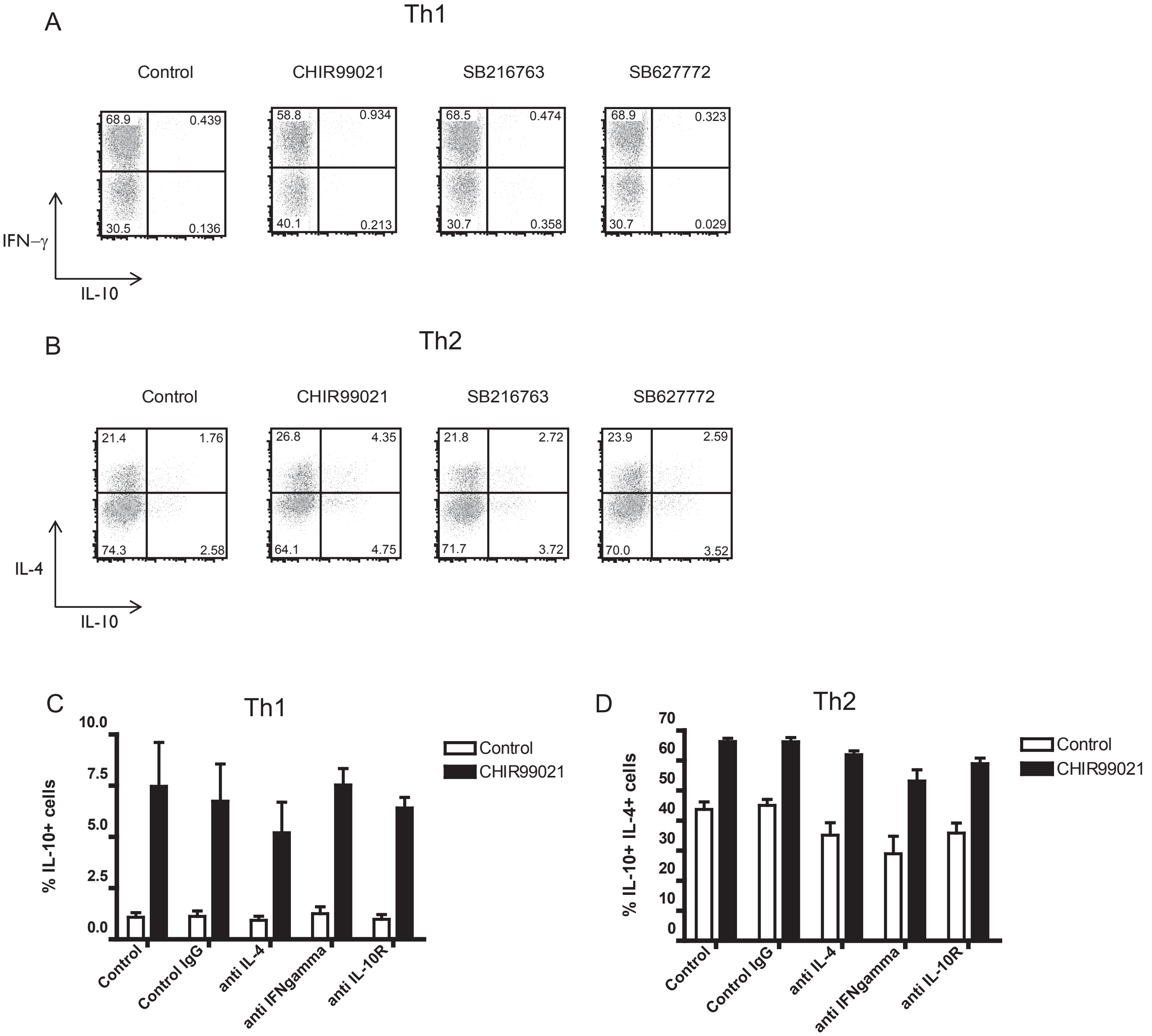

GSK3 inhibition induced IL-10 production is dependent on TCR stimulation but not on IL-4, IFNγ or IL-10 secretion.

Th1 (A) and Th2(B) polarised Tg4 cells were cultured with APCs in the presence of GSK3 inhibitors or vehicle control in the absence of MBP Ac1-9 peptide. IL-2 was added on day 3 of culture and on day 7 intracellular cytokine staining was carried out. Data are plots gated on live, $CD4^+$ cells and are representative of 4 (Th1) or 3 (Th2) independent experiments.

Th1 (A) and Th2(B) polarised $CD4^+$ cells were cultured on anti CD3/ anti CD28 coated plates in the absence/ presence of GSK3 inhibitor and no antibody or control IgG (HRPN; 10μg/ml), anti IL-4 (11B11; 10μg/ml), anti IFN-γ (XMG1.2; 25μg/ml) or anti IL-10R (1B1.3A; 10μg/ml).
On day 2 of culture cells were transferred to an uncoated plate and IL-2 added to the cultures on day 3. Intracellular cytokine staining was carried out on day 7 (C, D). Data shown are the mean and SEM from 4 (Th1) or 6 (Th2) independent experiments.

Supporting Information Figure 8

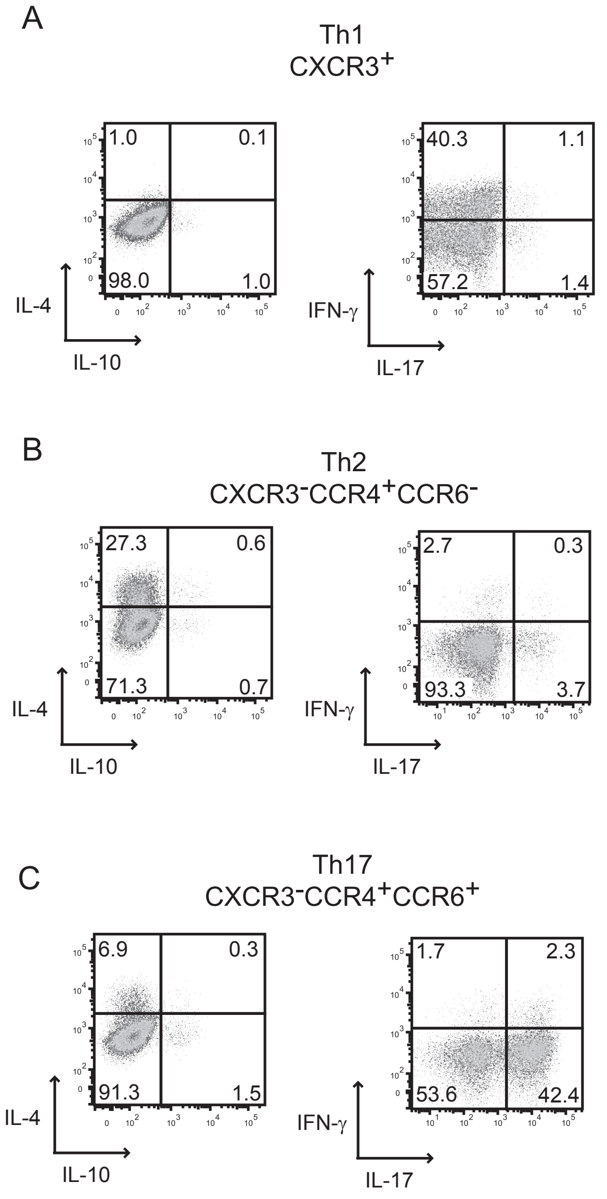

Cytokine production by human Th1, Th2 and Th17 cell subsets, flow cytometrically sorted based on chemokine receptor expression.

Human effector $CD4^+$ T cell subsets were isolated from healthy donors by flowcytometric sorting. Th1 cells ($CD4^+CXCR3^+$), Th2 cells ($CD4^+CXCR3^-CCR4^+CCR6^-$) or Th17 cells ($CD4^+CXCR3^-CCR4^+CCR6^+$) were cultured with autologous APC, anti-CD3 and IL-2. Intracellular cytokine staining was carried out on day 7. Dotplots show cytokine production by live, $CD4^+$ (A) Th1 cells, (B) Th2 cells or (C) Th17 cells. Data are representative of three experiments.

Supporting Information Figure 9

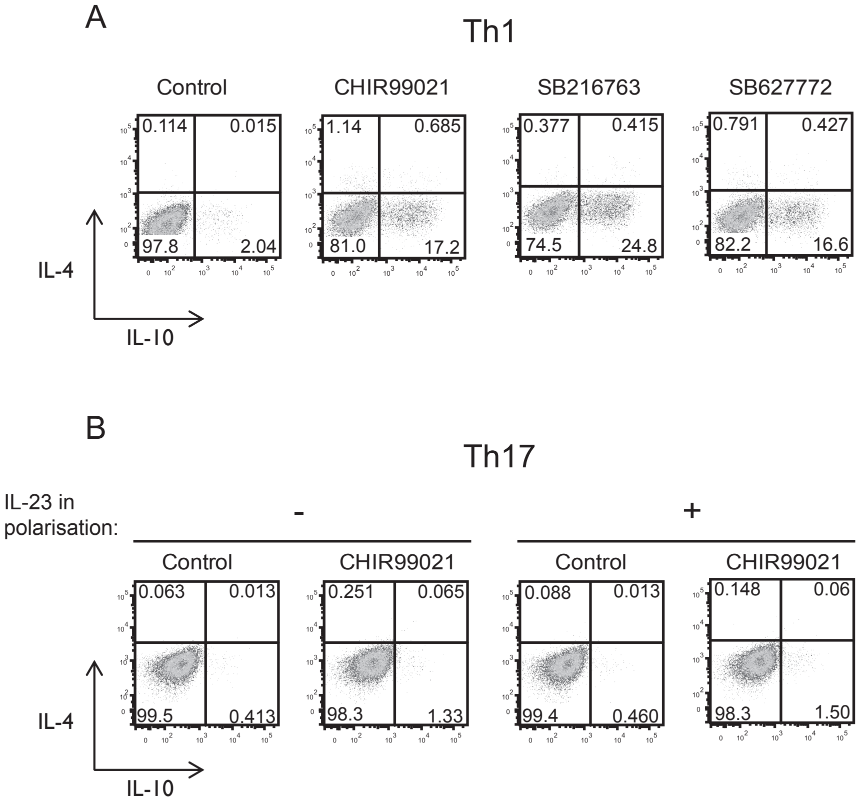

GSK3 inhibition does not promote IL-4 expression in Th1 and Th17 cells.

A. Tg4 splenocytes were cultured in the presence of peptide MBP Ac1-9 (10μg/ml) under Th1 polarising conditions. On day 7 of culture, Th1 cells were restimulated with APCs in the presence of GSK3 inhibitors or vehicle control as well as peptide MBP Ac1-9 (10μg/ml). IL-2 was added on day 3 of culture and intracellular cytokine staining for IL-4 and IL-10 carried out on day 7 of stimulation. Data are plots gated on live $CD4^+$ cells and are representative of 5 independent experiments.

B. Tg4 splenocytes were cultured in the presence of peptide MBP Ac1-9 (10μg/ml) under Th17 polarising conditions with or without the addition of IL-23 on day 3 as indicated.
On day 7 of culture, Th17 cells were restimulated with APCs in the presence of GSK3 inhibitors or vehicle control as well as peptide MBP Ac1-9 (10μg/ml). Intracellular cytokine staining for IL-4 and IL-10 was carried out on day 7 of stimulation. Data are plots gated on live $CD4^+$ cells and are representative of 2
independent experiments.

Supporting Information Figure 10

A

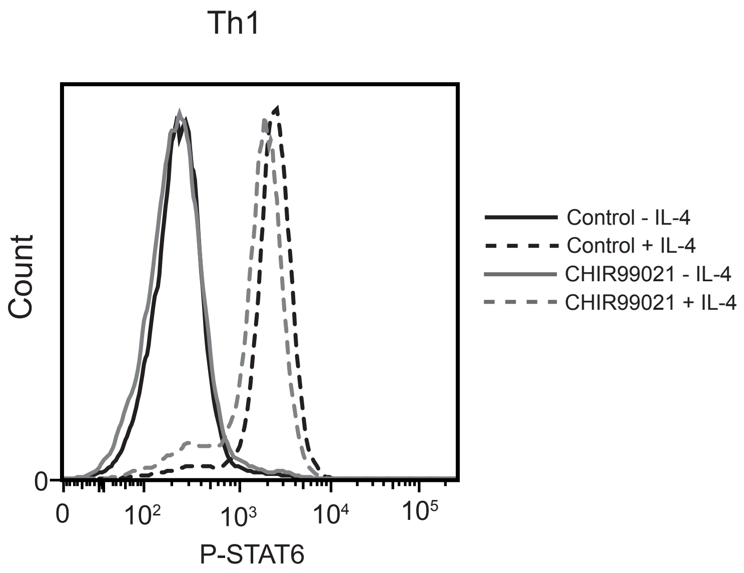

B

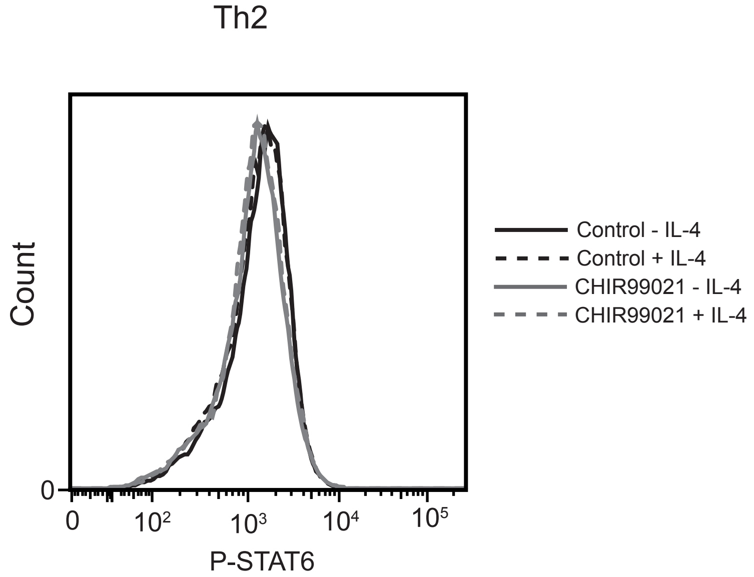

Phospho-STAT6 status of Th1 and Th2 cells is unaffected by culture with GSK3 inhibitors.

Th1 (A) and Th2 (B) polarised Tg4 cells were restimulated with APCs in the presence of GSK3 inhibitor CHIR99021 (2μM) or vehicle control and MBP Ac1-9. IL-2 was added on day 3 of culture and on day 7 live cells were stimulated with or without IL-4 (20ng/ml) for 15min at 37°C and stained with anti-CD4 FITC during this incubation.
Cells were fixed with 4% PFA on ice for 20min and permeabilised with 90% methanol at -20°C. Following permeabilisation, cells were stained for phospho-STAT6 (Y641) for 1 hour at room temperature and analysed by FACS. Data shown are gated on CD4 cells and are representative of three independent experiments.
